# Difficulty accessing crack pipes and crack pipe sharing among people who use drugs in Vancouver, Canada

**DOI:** 10.1186/1747-597X-6-34

**Published:** 2011-12-30

**Authors:** Lianping Ti, Jane Buxton, Evan Wood, Ruth Zhang, Julio Montaner, Thomas Kerr

**Affiliations:** 1 Faculty of Health Sciences, Simon Fraser University, 8888 University Drive, Burnaby, BC V5A 1S6, Canada; 2 British Columbia Centre for Excellence in HIV/AIDS, St. Paul's Hospital, 608-1081 Burrard Street, Vancouver, BC V6Z 1Y6, Canada; 3 Epidemiology Services, British Columbia Centre for Disease Control, 655 West 12th Avenue, Vancouver, BC V5Z 4R4, Canada; 4 School of Population and Public Health, University of British Columbia, 2206 East Mall, Vancouver, BC V6T 1Z3, Canada; 5 Department of Medicine, University of British Columbia, St. Paul's Hospital, 1081 Burrard Street, Vancouver, BC V6Z 1Y6, Canada

**Keywords:** crack cocaine smoking, injection drug use, harm reduction, sharing drug paraphernalia

## Abstract

**Background:**

Crack pipe sharing can increase health risks among people who use drugs, yet the reasons for sharing these pipes have not been well described. Therefore, we sought to identify the prevalence and correlates of crack pipe sharing among a community-recruited sample of people who use illicit drugs in Vancouver, a setting where crack pipes are provided at low or no cost.

**Findings:**

Data for this study were derived from two prospective cohorts of people who use drugs: the Vancouver Injection Drug Users Study (VIDUS) and the AIDS Care Cohort to evaluate Exposure to Survival Services (ACCESS). Multivariate logistic regression was used to identify factors independently associated with crack pipe sharing. Among 503 crack users, 238 (47.3%) participants reported having shared a crack pipe in the previous six months. Having acquired a mouthpiece in the last six months (adjusted odds ratio [AOR] = 1.91; 95% confidence interval [CI]: 1.31 - 2.79) and difficulty finding new pipes (AOR = 2.19; 95%CI: 1.42 - 3.37) were positively associated with pipe sharing. Binge drug use (AOR = 1.39; 95%CI: 0.96 - 2.02) was marginally associated with sharing pipes.

**Discussion:**

There was a high prevalence of crack pipe sharing in a setting where crack pipes are distributed at low or no cost. Difficulty accessing crack pipes was independently and positively associated with this behavior. These findings suggest that additional efforts are needed to discourage crack pipe sharing as well as increase access to crack pipes.

## Introduction

Crack cocaine use continues to be associated with various health-related harms. Injuries such as blisters, sores, and cuts on the lips and gums are common among people who smoke crack [1], and crack use has been associated with hepatitis C virus (HCV) and human immunodeficiency virus (HIV) infection [2,3]. There is evidence suggesting that injuries to the oral mucosa promote the transmission of HCV when crack smoking paraphernalia are shared between individuals with oral lesions [4]. However, in the case of HIV, the transmission pathways have not been determined and may reflect elevated syringe sharing or unsafe sex among crack users [2,5]. Other studies have pointed to crack use as a potential mode for the transmission of other infectious diseases including tuberculosis [6,7]. However, increasing access to crack pipes may reduce the frequency of injecting and by extension, blood-borne disease transmission among this population [8].

There is growing support for programs that facilitate access to sterile and safer crack smoking paraphernalia [8,9]. However, these programs remain controversial, and in Canada a number of these programs have been shutdown in response to concerns expressed by policy makers and the public [10,11]. Despite these developments and the high prevalence of crack cocaine use in settings throughout North America [2,12,13], little is known about why people who smoke crack continue to engage in risky behavior such as crack pipe sharing. Further, although past research has revealed that problems with access to sterile syringes can drive syringe sharing in settings where large needle exchange programs operate [14], little is known about the relationship between crack pipe access and crack pipe sharing. Therefore, we sought to investigate crack pipe sharing and access in Vancouver, Canada, a setting where crack smoking is prevalent and where crack pipes are provided at no or low cost.

## Background

Vancouver has experienced a massive growth in crack use over the past decade [12]. According to the 2010 Canadian Alcohol and Drug Use Monitoring Survey, the prevalence of crack cocaine use in BC (6.9%) is higher than in any other province [15]. A study in Vancouver demonstrated that among a sample of drug-using participants, daily crack use increased from 7.4% in 1996 to 42.6% in 2005. In the same study, crack use was independently associated with several characteristics, including sex work involvement and unstable housing [12]. Crack cocaine smoking has also been associated with the acquisition of HIV infection among people who inject drugs (IDU) in the city [2].

Locally, crack smoking paraphernalia, including Pyrex pipes, plastic mouthpieces and wooden push sticks, are provided through the BC Centre for Disease Control (BCCDC). A mouthpiece made of clear vinyl tubing is used as an attachment for crack pipes as a means of reducing risks for blood-borne disease transmission and other harms [16]. Wooden push sticks are used as an alternative to plastic syringe plungers, which are often used when packing a screen into a pipe [17]. Distributing wooden sticks helps reduce the likelihood that melted plastic is inhaled and also prevents the misuse and discarding of syringes [17]. Crack pipes in the form of hollow glass tubes are available at some harm reduction distribution sites in Vancouver at no or low cost, but are not universally available for free throughout the city. Local stores also sell crack pipes in areas where crack smokers live or congregate.

## Methods

Data for this study were derived from two prospective cohorts involving people who use drugs: the Vancouver Injection Drug Users Study (VIDUS) and the AIDS Care Cohort to evaluate Exposure to Survival Services (ACCESS). The methods for these studies have been previously described [18,19]. In brief, beginning in May 1996, participants were recruited through street outreach and self-referral in the Greater Vancouver Regional District. VIDUS eligibility criteria included having injected illicit drugs at least once in the previous month. ACCESS eligibility criteria included being HIV-positive, and having used illicit drugs other than cannabinoids in the previous month. At baseline visit and semi-annually thereafter, participants complete an interviewer-administered questionnaire and provide blood samples. The questionnaire elicits information about socio-demographic characteristics, drug use and other behavioral patterns, income-generation practices, engagement with medical and addiction treatment services, law enforcement encounters, and other experiences with the criminal justice system. At each study visit participants were provided with an honoraria ($20 CAD). The study has received ethics approval from the University of British Columbia/Providence Health Care Research Ethics Board. The present analyses were restricted to participants who reported smoking crack cocaine in the last six months, and were seen between December 2010 and May 2011. We selected the most recent follow-up period due to recent changes to the crack pipe distribution program in Vancouver. Specifically, we wanted to characterize current rates of sharing among this subpopulation in the wake of these programmatic changes. The collection of these data is being used to inform program development for crack users.

For the present analyses, we used univariate and multivariate logistic regression, with the outcome being having reported crack pipe sharing in the last six months. In this instance, crack pipe sharing referred to either crack pipe borrowing or lending. Variables considered included: median age, gender, living in the Downtown Eastside (DTES) of Vancouver (yes vs. no), frequency of crack use (≥ once per day vs. < once per day), having acquired a mouthpiece (yes vs. no), difficulty accessing crack pipes (yes/sometimes vs. no), smoking crack in public (always/usually vs. sometimes/occasionally/never), and binge use of non-injection drugs (yes vs. no). All behavioral variables refer to behaviors in the past six months. The variable "having acquired a mouthpiece" referred to obtaining a sterile mouthpiece from sites distributing crack pipe supplies. Additionally, the variable "difficulty accessing pipes" referred to whether participants found it difficult to obtain new crack pipes when they needed them. "Binge use of non-injection drugs" referred to whether the participant used non-injection drugs more than usual. To examine bivariate associations, we used the Pearson χ^2 ^test. Fisher's exact test was used when one or more cells contained values less than or equal to five. We then built a multivariate logistic regression model to identify independent predictors of crack pipe sharing by including all variables that were associated with the outcome at the *p *≤ 0.10 level in bivariate analyses. All *p*-values were two-sided. As a subanalysis, we asked participants to specify where they acquired their crack pipes.

## Results

In total, 503 drug users reporting crack cocaine smoking in the past six months participated in this study, including 181 (36.0%) females. These participants represented 61.1% of the total number of participants who were recruited into both VIDUS and ACCESS cohorts for this study period. The median age of participants was 46 years (range = 23 - 72 years). In total, 238 (47.3%) reported having shared a crack pipe in the last six months. As well, 63.6% were active injection drug users and 60.6% reported having been in alcohol or drug treatment in the past six months. As indicated in Table 1, factors significantly and positively associated with crack pipe sharing included having acquired a mouthpiece, difficulty accessing crack pipes, and binge use of non-injection drugs. Additionally, age was negatively associated with pipe sharing (all *p *< 0.10).

**Table 1:** Bivariate analyses of factors associated with crack pipe sharing among people who smoke crack cocaine (n = 503)

	Shared a crack pipe in the last six months n (%)			
**Characteristic**	**Yes** **238 (47.3)**	**No** **265 (52.7)**	**Odds Ratio (95% CI)**	***p - *value**

**Age**				
≥ 46 years old	116 (43.8)	149 (56.2)	0.74 (0.52 - 1.05)	0.09
< 46 years old	122 (51.3)	116 (48.7)		
**Gender**				
Female	84 (46.4)	97 (53.6)	0.94 (0.66 - 1.36)	0.76
Male	154 (47.8)	168 (52.2)		
**Living in DTES**				
Yes	157 (45.4)	189 (54.6)	0.78 (0.53 - 1.14)	0.20
No	81 (51.6)	76 (48.4)		
Frequency of crack use*				
≥ once per day	99 (51.6)	93 (48.4)	1.32 (0.92 - 1.89)	0.13
< once per day	139 (44.7)	172 (55.3)		
Acquired mouthpiece*				
Yes	162 (54.9)	133 (45.1)	2.12 (1.47 - 3.04)	< 0.01
No	76 (36.5)	132 (63.5)		
**Difficulty accessing pipes**				
Yes/Sometimes	78 (63.4)	45 (36.6)	2.38 (1.57 - 3.63)	< 0.01
No	160 (42.1)	220 (57.9)		
Smoke in public*				
Always/Usually	53 (52.0)	49 (48.0)	1.26 (0.82 - 1.95)	0.29
Sometimes/Occasionally/Never	185 (46.1)	216 (53.9)		
Binge drug use (non-injection)*^1^				
Yes	127 (53.8)	109 (46.2)	1.65 (1.15 - 2.36)	< 0.01
No	106 (41.4)	150 (58.6)		

DTES: Downtown Eastside of Vancouver

CI: confidence interval

*Activities/events in the last six months

^1^Note that counts for binge drug use do not add up to n = 503 due to 11 missing responses (n = 492).

As indicated in Table 2, in multivariate analyses, factors that remained positively associated with crack pipe sharing included: having acquired a mouthpiece and difficulty accessing crack pipes (*p *< 0.05). Participants reported access to crack pipes through a variety of sources, including: street (new) (39.4%), corner store (29.8%), a drug user-run organization (25.6%), and local health programs (8.2%). As well, a number of participants (9.9%) reported acquiring used pipes from the street.

**Table 2:** Multivariate logistic regression analyses of factors associated with crack pipe sharing among people who smoke crack cocaine (n = 503)^1^

Variable	Adjusted Odds Ratio (AOR)	95% CI	*p - *value
**Age**			
(≥ 46 years vs. < 46 years)	0.80	(0.56 - 1.17)	0.25
Acquired mouthpiece*			
(Yes vs. No)	1.91	(1.31 - 2.79)	< 0.01
**Difficulty accessing pipes**			
(Yes/Sometimes vs. No)	2.19	(1.42 - 3.37)	< 0.01
Binge drug use (non-injection)*			
(Yes vs. No)	1.39	(0.96 - 2.02)	0.08

CI: confidence interval

*Activities/events in the last six months

^1^Χ^2 ^(Likelihood Ratio Test) = 35.68; p-value = < 0.01; R^2 ^= 0.07

## Discussion

In the present analyses, we found a high rate of crack pipe sharing among illicit drug users in Vancouver, with just under 50% of participants reporting crack pipe sharing in the previous six months. Those participants who reported sharing crack pipes were more likely to report having recently acquired a mouthpiece and to have experienced difficulty accessing pipes.

Our findings are consistent with previous studies reporting high rates of crack pipe sharing among people who smoke crack [8]. Although there is limited evidence concerning the impact of programs that target crack smokers, a study in Ottawa, Canada reported positive outcomes from distributing crack-smoking paraphernalia, including a decline in the frequency of pipe sharing [8]. Given the health risks associated with pipe sharing and our finding that difficulty accessing pipes was associated with sharing, increasing access to crack pipes has potential to reduce the transmission of infectious diseases as well as injuries to the oral cavity via use of makeshift devices [4,6].

Recent efforts have been made by BCCDC to distribute mouthpieces province-wide. Mouthpiece distribution provides a point of engagement for crack smokers who may not inject since many may not have been connected with harm reduction services otherwise [16]. In the event where crack pipes are unavailable, and sharing a pipe becomes inevitable, mouthpieces can alleviate the risk of injuries and infections associated with pipe sharing. Consistently, our findings showed a positive association between mouthpiece acquisition and sharing crack pipes, indicating that many people who smoke crack are practicing safer smoking methods. However, it is unclear whether individuals are using their own mouthpiece when sharing pipes. Given the lack of evidence describing the negative health consequences from sharing a mouthpiece, future research should focus on examining the different health risks between individuals who share and do not share mouthpieces.

This study has limitations. First, because of the cross-sectional design, determining a temporal relationship between exposure and outcome is not possible. Second, both VIDUS and ACCESS cohorts are community-recruited non-randomized samples of IDU and HIV-positive drug users and therefore our findings may not be generalizable to all crack cocaine users in local or other settings. Third, from this study, it is not clear whether the participants are using their own mouthpiece, sharing a mouthpiece, or not using a mouthpiece at all when they are sharing crack pipes. Lastly, the data collected were self-reported and may be subject to reporting biases.

In summary, we found that crack pipe sharing is common among people who smoke crack in Vancouver. Difficulty accessing pipes and having acquired a mouthpiece were positively associated with crack pipe sharing. Although many cities worldwide, including Vancouver, have succeeded in providing harm reduction paraphernalia for IDU, similar services for people who smoke crack are not as readily available [20]. Our findings suggest that additional crack pipe distribution efforts are needed to address the risks and harms associated with crack use.

## Competing interests

Dr. Julio Montaner has received grants from, served as an ad hoc advisor to, or spoke at various event sponsored by: Abbott, Argos therapeutics, Bioject Inc., Boehringer Ingelheim, BMS, Gilead Sciences, GlaxoSmithKline, Hoffmann-La Roche, Janssen-Ortho, Merck Frosst, Pfizer, Schering, Serono Inc., TheraTechnologies, Tibotec, and Trimeris. Other authors declare that they have no other competing interests.

## Authors' contributions

The specific contributions of each author are as follows: LT and TK were responsible for study design; RZ conducted the statistical analyses; LT prepared the first draft of the analysis; All authors provided critical comments on the first draft of the manuscript and approved the final version to be submitted.

## References

[B1] Porter J Bonilla L Crack Users' Crack Lips: An Additional HIV Risk Factor Am J Public Health 1993 83 10 1490 1491 8214248 10.2105/ajph.83.10.1490-a PMC1694867

[B2] DeBeck K Kerr T Li K Fischer B Buxton J Montaner J Wood E Smoking of crack cocaine as a risk factor for HIV infection among people who use injection drugs CMAJ 2008 181 9 585 589 10.1503/cmaj.082054 PMC2764753 19841052

[B3] Nyamathi AM Dixon EL Robbins W Smith C Wiley D Leake B Longshore D Gelberg L Risk Factors for Hepatitis C Virus Infection Among Homeless Adults J Gen Intern Med 2002 17 134 143 10.1046/j.1525-1497.2002.10415.x 11841529 PMC1495011

[B4] Tortu S McMahon JM Pouget ER Hamid R Sharing of Noninjection Drug-Use Implements as a Risk Factor for Hepatitis C Subst Use Misuse 2004 39 2 211 224 10.1081/JA-120028488 15061559

[B5] Edlin BR Irwin KL Faruque S McCoy CB Word C Serrano Y Inciardi JA Bowser BP Schilling RF the Multicenter Crack Cocaine and HIV Infection Study Team Intersecting epidemics - Crack cocaine use and HIV infection among inner-city young adults N Engl J Med 1994 331 21 1422 1427 10.1056/NEJM199411243312106 7969281

[B6] Gardy JL Johnston JC Ho Sui SJ Cook VJ Shah L Brodkin E Rempel S Moore R Zhao Y Holt R Varhol R Birol I Lem M Sharma MK Elwood K Kones SJ Brinkman FS Brunham RC Tang P Whole-Genome Sequencing and Social-Network Analysis of a Tuberculosis Outbreak N Engl J Med 2011 364 730 739 10.1056/NEJMoa1003176 21345102

[B7] McElroy PD Rothenberg RB Varghese R Woodruff R Minns GO Muth SQ Lambert LA Ridzon R A network-informed approach to investigating a tuberculosis outbreak: implications for enhancing contact investigations Int J Tuberc Lung Dis 2003 7 12 Suppl 3 S486 493 14677842

[B8] Leonard L DeBuneis E Pelude L Medd E Birkett N Seto J "I inject less as I have easier access to pipes": Injecting, and sharing of crack-smoking materials, decline as safer crack-smoking resources are distributed Int J Drug Policy 2008 19 255 264 10.1016/j.drugpo.2007.02.008 18502378

[B9] British Columbia Harm Reduction Strategies and Services Best Practices for British Columbia's Harm Reduction Supply Distribution Program 2008 Vancouver: BC Centre for Disease Control

[B10] Bailey I Rethinking crack kits for addicts The Globe and Mail 2008

[B11] Dormer D Province shuts down free crack pipe program Calgary Sun 2011

[B12] United Nations Office on Drugs and Crime World Drug Report 2011 2011 Vienna: United Nations Office on Drugs and Crime

[B13] Werb D DeBeck K Kerr T Li K Montaner J Wood E Modelling crack cocaine use trends over 10 years in a Canadian setting Drug Alcohol Rev 2010 29 271 277 10.1111/j.1465-3362.2009.00145.x 20565519 PMC4643832

[B14] Marshall BD Shoveller JA Wood E Patterson TL Kerr T Difficulty Accessing Syringes Mediates the Relationship Between Methamphetamine Use and Syringe Sharing Among Young Injection Drug Users AIDS Behav 2010 10.1007/s10461-010-9876-8 PMC3180618 21197598

[B15] Health Canada Canadian Alcohol and Drug Use Monitoring Survey 2010 Ottawa, ON: Office of Drugs and Alcohol Research and Surveillance Controlled Substances and Tobacco Directorate, Health Canada

[B16] British Columbia Harm Reduction Strategies and Services Crack Pipe Mouthpieces: Questions and Answers 2010 Vancouver: BC Centre for Disease Control

[B17] British Columbia Harm Reduction Strategies and Services Crack Pipe Push Sticks: Questions and Answers 2010 Vancouver: BC Centre for Disease Control

[B18] Buxton J Kerr T Qi J Montaner J Wood E Public crack cocaine smoking and willingness to use a supervised inhalation facility: implications for street disorder Subst Abuse Treat Prev Policy 2011 6 4 10.1186/1747-597X-6-4 PMC3049126 21345231

[B19] Palepu A Milloy M Kerr T Zhang R Wood E Homelessness and Adherence to Antiretroviral Therapy among a Cohort of HIV-Infected Injection Drug Users J Urban Health 2011 88 3 545 555 10.1007/s11524-011-9562-9 21409604 PMC3126933

[B20] Haydon E Fischer B Crack Use As a Public Health Problem in Canada: Call for an Evaluation of 'Safer Crack Use Kits' Can J Public Health 2005 96 3 185 188 15913081 10.1007/BF03403687 PMC6976094

